# A Double Challenge for Fish: The Combined Stress of Warming and Pharmaceuticals in Aquatic Systems

**DOI:** 10.3390/jox15060190

**Published:** 2025-11-08

**Authors:** Tiago Lourenço, Maria João Rocha, Eduardo Rocha, Tânia Vieira Madureira

**Affiliations:** 1 Group of Animal Morphology and Toxicology, Interdisciplinary Centre of Marine and Environmental Research (CIIMAR/CIMAR), University of Porto, Terminal de Cruzeiros do Porto de Leixões, Av. General Norton de Matos s/n, 4450-208 Matosinhos, Portugal; up201707353@edu.icbas.up.pt (T.L.); mjrocha@icbas.up.pt (M.J.R.); 2 Laboratory of Histology and Embryology, Department of Microscopy, ICBAS—School of Medicine and Biomedical Sciences, University of Porto, Rua Jorge Viterbo Ferreira 228, 4050-313 Porto, Portugal

**Keywords:** aquatic toxicology, fish physiology, global warming, pharmaceutical pollution, temperature-pharmaceutical interactions

## Abstract

Aquatic ecosystems are increasingly threatened by multiple anthropogenic stressors, notably climate change and pollution by pharmaceuticals. Global warming is predicted to raise water temperatures by 2–5 °C by the end of the century. As ectotherms, fish are particularly vulnerable due to limited thermal tolerance and temperature-dependent physiology. Pharmaceuticals are introduced into aquatic systems at concentrations ranging from ng·L^−1^ to µg·L^−1^, including widely prescribed classes such as antibiotics, hormones, analgesics, antifungals, and neuropsychiatric drugs. This narrative review synthesizes experimental evidence on the interactive effects of warming and pharmaceutical exposure in fish. Thirty-nine peer-reviewed studies published since 2005 were analyzed. The findings indicate that higher temperatures often exacerbate pharmaceutical-induced toxicity, altering oxidative stress, metabolism, reproduction, and behavior. Antibiotic-focused studies showed temperature-dependent acceleration of absorption, distribution, metabolism, and excretion, with shorter half-lives and reduced tissue persistence at higher temperatures. Estrogenic hormones and antifungals have been shown to interact with thermal regimes, disrupting reproductive physiology and skewing sex ratios, particularly in species exhibiting temperature-dependent sex determination. Neuropsychiatric drugs exhibited altered uptake and metabolism under warming conditions, resulting in increased brain bioaccumulation and behavioral alterations affecting ecological fitness. Analgesics and anti-inflammatories remain understudied despite their widespread use, with evidence suggesting synergistic effects on oxidative stress at elevated temperatures. Significant research gaps persist regarding chronic exposures, early developmental stages, ecologically relevant temperature scenarios, and underrepresented or absent drug classes, such as hypolipidemic drugs. Ultimately, broader and integrated approaches are needed to better understand and predict the ecological risks of pharmaceutical pollution in a warming world.

## 1. Introduction

Aquatic ecosystems are being increasingly impacted by anthropogenic activities, namely those related to climate change, including global warming. A direct consequence of the latter is the increase in water temperatures, which are expected to rise by 2 to 5 °C by the end of the century [1]. As ectothermic organisms, fish generally have body temperatures similar to the environmental temperature [2], meaning that their physiological processes are strongly influenced by water temperature [3].

The susceptibility of fish to warm stress is influenced mainly by their temperature tolerance range, which is often relatively narrow—particularly during critical life stages, such as embryogenesis and spawning [4]. Although fish tend to seek their optimal temperature range [5,6], physical barriers in their habitats can limit movement to more suitable thermal environments [2]. Within this context, riverine systems may be particularly vulnerable not only to pollution stressors, such as the highly toxic heavy metals [7,8], but also to temperature increases driven by climate change, which are projected to be more pronounced in rivers than in marine environments [9]. The temperature effects have already been reported to influence many aspects of fish physiology [10,11]. This is particularly evident in growth regulation, where changes from optimal temperatures tend to reduce growth in Atlantic salmon (*Salmo salar*) [12] or the guppyfish (*Poecilia reticulata*) [13]. Higher temperatures have also been shown to alter hepatic glycogen and catalase levels in female zebrafish (*Danio rerio*) [14] and to increase vitellogenin mRNA expression in the liver of males [15]. Beyond their direct effects on growth and reproduction, rising temperatures can also influence fish respiratory performance [16], immune function [17], energy metabolism [18] and behavior [19]. Over time, these physiological stresses and adaptations can increase disease susceptibility and shift sex ratios, ultimately threatening individual fitness and population maintenance [17,18,19].

**Figure 1:**
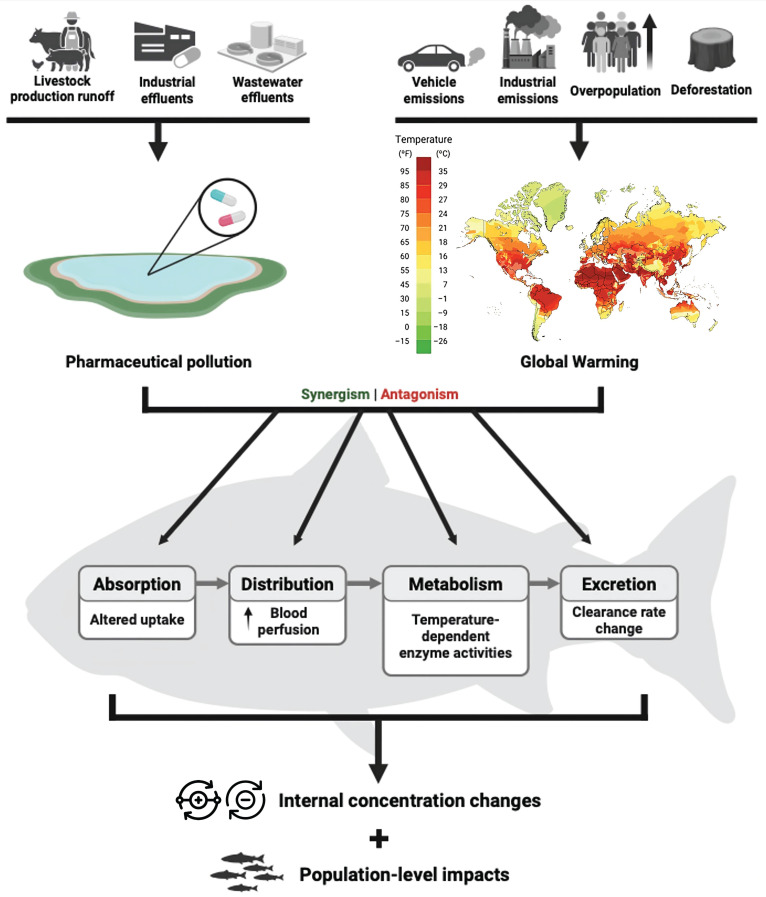
Sources and pharmacokinetic impacts of pharmaceutical pollution and global warming in fish.

Rising temperatures are not the only threat to aquatic ecosystems; pharmaceutical pollution is also a significant emerging stressor in these systems [20,21] (Figure 1). The consumption of pharmaceuticals has risen sharply in recent decades, driven by the rapid development of new drugs [22]. Consequently, these substances often enter aquatic environments through various pathways, with wastewater treatment plant (WWTP) effluents being one of the most frequently cited sources [23]. Among the most commonly prescribed medications, analgesics, antibiotics, and anti-inflammatories have been detected in distinct water matrices at ng·L^−1^ concentrations [22]. Numerous studies worldwide have focused on quantifying these pharmaceutical classes, detecting them in diverse water bodies across regions such Europe (e.g., [24,25,26,27,28,29,30]), North America (e.g., [31,32,33]), Central and South America (e.g., [34,35]), East and Southeast Asia (e.g., [36,37,38,39,40,41,42,43]) and Africa (e.g., [44,45,46,47]). Other pharmaceutical classes, such as antidepressants and lipid-lowering agents, are also commonly found in aquatic environments [28,48,49,50]. Pharmaceuticals are designed to be effective at low concentrations, which means that even the low levels continuously entering aquatic systems can affect non-target organisms [51]. Since pharmaceuticals interact with specific molecular targets, their adverse effects are more likely to impact targets that are evolutionarily conserved [52]. This is indeed the case for many aquatic species, such as zebrafish, which presented orthologs to over 1100 genetic human drug targets [51]. 

Interactions between xenobiotics and temperature are much less studied than the effects of each individual stressor. In recent years, there has been a shift towards the testing of combinations of stressors, such as high-temperature scenarios and pesticide [53,54] or heavy metal exposures [55,56,57,58]. Nevertheless, studies investigating the combined effects of temperature and pharmaceutical exposures remain rather limited despite being justifiable. Temperature influences the toxicokinetic of xenobiotics in fish by modulating absorption, distribution, metabolism, and excretion [59,60]. Warmer water generally increases ventilation, cardiac output, and gastrointestinal perfusion, thereby enhancing the uptake rates of pollutants [61,62]. Temperature-induced alterations in biotransformation enzyme activity (e.g., cytochrome P450s, UDP-glucuronosyltransferases) may either accelerate or inhibit metabolism depending on the compound and the species involved [63]. For example, higher temperatures have been shown to modify the pharmacokinetics of enrofloxacin and its metabolite ciprofloxacin in largemouth bass (*Micropterus salmoides*) [64] and in black rockfish (*Sebastes schlegelii*) [65], as well as the elimination of oxolinic acid in black rockfish [66], resulting in altered absorption, metabolic conversion, and clearance rates. These findings illustrate that warming can disrupt toxicokinetic profiles, potentially increasing internal exposure to pharmaceuticals or their metabolites even when external concentrations remain constant [67,68]. Given the actual environmental scenario of ubiquitous pharmaceutical pollution across aquatic compartments, global warming represents an additional threat to wild fish populations (Figure 1).

With this in mind, a literature search was conducted for this review using Scopus, PubMed and Web of Science, with the search restricted to studies published from 2005 onward. Search terms included combinations of keywords such as “pollutant(s)”; “pharmaceutical(s)”; “contaminant(s)”; “water temperature”; and “fish(es)”. The resulting peer-reviewed papers were screened and included if they used fish species (freshwater or marine) at any life stage, assessed simultaneous exposure to elevated temperatures and pharmaceuticals, and were experimental investigations (laboratory- or field-based) (Supplementary Table S1). Studies focusing on other pollutant classes (e.g., persistent organic pollutants), using different experimental models, or not including water temperature as a stressor were excluded. The primary objective of this review is to provide a comprehensive synthesis of current knowledge on the combined effects of warming scenarios and pharmaceutical exposure on fish. It also aims to draw attention to critical knowledge gaps—such as fish species representation, overlooked pharmaceutical classes, and types of exposures (e.g., acute, chronic)—that might help the design of upcoming experimental studies and the establishment of priority environmental regulations and conservation strategies.

## 2. Analgesics and Anti-Inflammatories

Analgesic and anti-inflammatory pharmaceuticals are among the most widely consumed drugs and, therefore, one of the most frequently detected in aquatic environments [22]. Despite this, to our knowledge, only four studies have evaluated the possible harmful effects of these compounds on fish when combined with elevated temperatures (Table 1). Boreham et al. [69] showed that a 72 h waterborne exposure to paracetamol (2 and 3 mM) and diclofenac (0.7, 1.4, 2 and 2.7 µM) can cause oxidative stress in transgenic zebrafish larvae, under two temperature regimes: 28 °C (optimal rearing temperature) and 33 °C, used to represent the predicted 5 °C increase caused by global warming. The larvae were EpRE:mCherry transgenic, exhibiting fluorescence in the tissues with oxidative stress response, as the EpRE is a promoter region for antioxidative pathways [69]. While paracetamol could induce oxidative stress at 28 °C, the effects were significantly stronger at the higher temperature (33 °C). In contrast, diclofenac did not induce oxidative stress at 28 °C, but, at 33 °C, it was the more potent of the two drugs, causing greater oxidative damage than paracetamol (Table 1). In our view, the observed responses to paracetamol and diclofenac suggest that temperature-dependent changes in toxicokinetic are compound-specific. Moreover, the enhanced effects of both pharmaceuticals under a high-temperature regime may be linked with impaired detoxification processes that fish can undergo at high temperature [63], which reduced metabolic efficiency and increased internal exposures. Similar effects were observed in Japanese medaka (*Oryzias latipes*) larvae exposed to the analgesic acetaminophen, at 50 and 150 mg·L^−1^, under optimal (25 °C), higher (30 °C) and lower (15 °C) temperature regimes [70]. 

After a 4-day exposure, liver vacuolization in medaka larvae increased in a temperature-dependent manner and was further exacerbated by co-exposure with acetaminophen (Table 1). In the context of global warming, this pattern suggests that higher water temperatures could amplify toxic effects, thereby raising the risk to aquatic organisms. Interestingly, certain physiological responses to warming, such as the increase in cellular ATP levels to support the enhanced metabolic demands [10,11], were suppressed when Japanese medaka larvae were simultaneously exposed to acetaminophen and elevated temperatures [70]. The increase in ATP demand at higher temperatures is dependent on mitochondrial efficiency, which tends to be reduced under hyperthermic stress [10,71]. This reduction in mitochondrial efficiency is often linked to an insufficient oxygen supply [11,72], which in turn can be associated with heart failure and reduced oxygen transportation capacity [71]. An increase in red blood cell count was observed in Japanese medaka larvae exposed to 50 mg·L^−1^ of acetaminophen at 30 °C, likely as a compensatory response to reduced oxygen availability. Yet, when the acetaminophen concentration was increased to 150 mg·L^−1^, at the same temperature, the effect was suppressed [70]. These impacts suggest that co-exposure to pharmaceuticals and thermal stress may impair physiological responses to warming environments. 

In contrast, two other findings diverge from this general trend of enhanced effects of analgesics and anti-inflammatories under elevated temperatures. An example is the study conducted by González-Mira et al. [73] on juvenile Senegalese sole (*Solea senegalensis*) by administration of ibuprofen via intraperitoneal (IP) injection, at pharmacologically relevant concentrations of 10 mg·kg^−1^, at either 15 °C or 20 °C (temperatures selected with a 5 °C interval to represent the global warming predictions). The study evaluated the activity of several hepatic biotransformation enzymes from the cytochrome P450 family to gain a better understanding of ibuprofen metabolism in fish and to identify potential biomarkers of exposure [73]. The activity of uridine-diphosphoglucuronosyltransferase (UDPGT) was more strongly inhibited by ibuprofen at 20 °C (Table 1). In contrast, the activity of 7-benzyloxy-4-trifluoromethylcoumarin-O-debenzyloxydase (BFCOD), which is associated with phase I biotransformation of xenobiotics, was only affected by ibuprofen under the lower temperature (15 °C). Another study using the same experimental set-up (ibuprofen at 10 mg·kg^−1^ via IP injection and temperature regimes of 15 and 20 °C) evaluated ibuprofen metabolites in bile as well as hepatic enzyme activity [74]. While no temperature-dependent differences in ibuprofen metabolites in bile were found, the activity of the phase I biotransformation enzymes, including carboxylesterases and CYP-mediated enzymes, was only detected at 15 °C and not at 20 °C (Table 1). Although the effects are reduced or absent at warmer temperatures, previous findings [73,74] suggested that this reduction is due to decreased biotransformation and detoxification capacity resulting from lower enzymatic activity or responsiveness. As such, and because enzymes become strained outside their optimal temperature range [75], biotransformation processes can be rate-limited at higher temperatures in fish.

**Table 1:** Summary of experimental conditions and main effects of the combined fish exposure to temperature and analgesics or anti-inflammatories.

Therapeutic Class	Species	Life Stage	Pharmaceutical(s)	Dose	Temperature (°C)	Exposure Period	Administration	Effects	Reference
Analgesic	*Danio rerio*	Larvae	Paracetamol	1; 2; 3 mM	28; 33	72 h	Water	Oxidative stress (OS) elevated at 33 °C, particularly in the liver and gastrointestinal tract.	[69]
								Paracetamol uptake increased at 33 °C	
Anti-inflammatory			Diclofenac	0.7; 1.4; 2.0; 2.7 µM				No OS at 28 °C.	
								Became the most potent OS-induced pharmaceutical at 33 °C.	
Analgesic	*Oryzias latipes*	Larvae	Paracetamol	50; 150 mg·L^−1^	15; 25; 30	4 days	Water	Increased body length.	[70]
								Decrease heart rate at 50 mg·L^−1^ and high temperature and at 150 mg·L^−1^.	
								Increased ATP content at 25 and 30 °C (50 mg·L^−1^).	
								Suppressed red blood cell (RBC) count at 30 °C (150 mg·L^−1^).	
								Temperature-dependent liver vacuolization enhanced by acetaminophen exposure.	
Anti-inflammatory	*Solea senegalensis*	Juvenile	Ibuprofen	10 mg·kg^−1^	15; 20	48 h	Intraperitoneal injection	CYP and carboxylesterase activities were traceable only at 15 °C.	[74]
Anti-inflammatory	*Solea senegalensis*	Juvenile	Ibuprofen	10 mg·kg^−1^	15; 20	48 h	Intraperitoneal injection	Decreased plasma lactate at both temperatures.	[73]
								Increased 7-benzyloxy-4-trifluoromethylcoumarin-O-debenzyloxydase (BFCOD) activity at 15 °C.	
								Greater suppression of uridine-diphosphoglucuronosyltransferase (UDPGT) activity decreased more at 20 °C.	

## 3. Antibiotics and Antifungals

Antibiotics are ubiquitously found in aquatic environments and are frequently detected at ng·L^−1^ concentrations [24,76], and occasionally at µg·L^−1^ levels [32]. Due to their widespread occurrence as environmental pollutants, antibiotics have received considerable attention regarding their toxic effects on fish. For example, erythromycin induced oxidative stress in the liver and gills of gilthead seabream (*Sparus aurata*) [77], oxidative stress and antioxidant suppression caused by oxytetracycline and sulfamethoxazole in Nile tilapia (*Oreochromis niloticus*) [78], and histopathological changes in the heart of zebrafish exposed to a mixture of β-diketone antibiotics [79]. Despite this, studies assessing the effects of antibiotics in combination with temperature remain limited. Most are pharmacokinetic investigations evaluating a single antibiotic concentration under distinct temperatures, typically representing seasonal variations [61,62,80,81,82,83,84,85,86,87] (Table 2). These studies used different species (Table 2), such as crucian carp (*Carassius auratus gibelio*) [62,85,86] or Nile tilapia [61,83], and distinct antibiotics, including tetracycline [87], amoxicillin [83] and florfenicol [81]. Exposure periods are generally short, ranging from a single antibiotic administration [62,85] up to 15 days [87] (Table 2). Despite methodological differences, these studies consistently showed that higher temperatures accelerated antibiotic pharmacokinetics in fish, resulting in shorter drug half-lives, lower peak concentrations, and a faster time to reach peak concentrations (Table 2). Moreover, although higher temperatures accelerate drug absorption and elimination, the metabolite formation rate and tissue distribution may also change, altering the internal dose and persistence of active or toxic metabolites [64,65]. A distinct study evaluated the toxicity of cefalexin at 20 °C and 25 °C using juveniles of the common goby (*Pomatoschistus microps*) as the model species. The antibiotic showed increased toxicity at the higher temperature (Table 2), as demonstrated by the lower EC50 of cefalexin [88]. Moreover, at 25 °C, all cefalexin concentrations caused mortality rates of up to 33% and reduced predatory performance, whereas at 20 °C, only the highest concentration (10 mg·L^−1^) induced mortality [88]. These findings suggest that higher temperatures might overload metabolic and excretory pathways, increase internal doses and toxicity and lead to such exacerbated effects. The oxidative stress and DNA damage seen in zebrafish at higher temperatures may be explained by a similar process [68].

Temperature-driven pharmacokinetic changes may impair key physiological functions, including immune competence, reproduction, and energy metabolism, by altering the duration and magnitude of antibiotic exposure [9,18]. At population level, these functional disruptions may be translated into increased mortality, and reduced growth and fertility, which could shift population structures and fitness [4].

Despite being used to treat fungal infections in humans, antifungal pharmaceuticals have been shown to disrupt reproductive pathways in fish [89,90,91]. For example, clotrimazole belongs to the azole fungicide family, which is known to interfere with cytochrome P-450 enzymes [92], including aromatase [93] and, consequently, with estrogen synthesis and sexual differentiation. In gonochoristic fish, sexual differentiation can be strongly influenced by environmental temperature, which can bias development towards male or female phenotypes [94], making this physiological process particularly vulnerable to climate change and endocrine disruption. In this context, Brown et al. [95] investigated how the combined exposure to clotrimazole and elevated temperatures affects sexual differentiation in zebrafish. The study tested two nominal concentrations of clotrimazole (2 µg·L^−1^ and 10 µg·L^−1^) at 28 °C—the typical spawning temperature of zebrafish—and at 33 °C, representing a 5 °C increase projected under the worst-case global warming scenario (Table 2). Zebrafish exposed to high concentrations of clotrimazole showed a clear bias towards male differentiation at both temperatures, with the effect being stronger at 33 °C [95] (Table 2). Even at the lower concentration (2 µg·L^−1^), a male-biased sex ratio was observed—but only at 33 °C (Table 2). These results expose risks of population-level impacts, as skewed sex ratios are a major demographic stressor. Persistent male bias lowers genetic diversity, decreases the size of the effective breeding population, and might cause reproductive collapse if females tend to become limiting [4,96].

The enhanced effects of antifungals at higher temperatures were also evident in their increased potential for bioaccumulation, such as in Atlantic croaker (*Micropogonias undulatus*) exposed to triclosan, a widely used antimicrobial and antifungal agent, under two temperature regimes: 26 °C (average summer temperature) and 29 °C (a predicted warming scenario) [97]. After 10 days of consuming a diet containing 50 mg·kg^−1^ triclosan, Atlantic croaker exhibited higher bioaccumulation capacity at the higher temperature (29 °C) (Table 2) [97]. This is another illustration of how temperature can alter toxicokinetic, particularly by influencing bioaccumulation dynamics. Interestingly, the same study found no temperature-dependent interaction with triclosan exposure for other endpoints, such as nerve reflexes or food intake [97], contradicting the general trend of increased pharmaceutical effects under warming conditions.

**Table 2:** Summary of methodology and effects of combined exposure to temperature and antibiotic or antifungal pharmaceuticals.

Therapeutic Class	Species	Life Stage	Pharmaceutical(s)	Dose	Temperature (°C)	Exposure Period	Administration	Effects	Reference
Antibiotic	*Micropterus salmoides*	Juvenile/Adult	Enrofloxacin	20 mg·kg^−1^	17; 22; 27	5 days	Oral gavage	Faster metabolization of antibiotics at 27 °C.	[64]
			Ciprofloxacin					Antibiotics had a shorter liver half-life at 27 °C.	
Antibiotic	*Oryzias malastigma*	Embryo	Florfenicol	0.5; 10; 100; 500; 1000 µg·L^−1^	20; 25; 30	21 days	Water	Hatching success was lowest at 30 °C with florfenicol at 10 and 100 µg.L^−1^.	[98]
								Upregulation of caspase-3 and caspase-9 at 30 °C.	
								Glutathione peroxidase activity was inhibited at 25 and 30 °C.	
Antibiotic	*Danio rerio*	Juvenile	Trimethoprim	30 µg·L^−1^	26; 28; 32	28 days	Water	Trimethoprim at 32 °C increased acetylcholinesterase activity.	[68]
								The antibiotic mixture increased glutathione peroxidase and superoxide dismutase activity.	
			Sulfamethoxazole	150 µg·L^−1^				Both antibiotics induced DNA strand breaks that were more pronounced at 32 °C.	
Antibiotic	*Sebastes schlegelii*	Adult	Oxolinic acid	30 mg·kg^−1^	13; 22	Single dose	Oral gavage	Maximum plasma concentration was higher at 22 °C	[66]
								Clearance of oxolinic acid was reduced at 22 °C.	
Antibiotic	*Sebastes schlegelii*	Juvenile	Enrofloxacin	10 mg·kg^−1^	13; 22	Single dose	Oral gavage	Maximum plasma concentration was lower at 22 °C.	[65]
			Ciprofloxacin					Elimination was slower at 22 °C.	
Antibiotic	*Oreochromis niloticus*	Juvenile	Amoxicillin	40 mg·kg^−1^	25; 30	5 days	Oral gavage	Detected concentrations of amoxicillin in muscle were higher at 25 °C on days 1-, 3- and 5 post treatment.	[83]
Antibiotic	*Salmo salar*	Juvenile	Tetracycline	100 mg·kg^−1^		15 days	Food	Tetracycline plasma concentration was lowest in fish kept at 20 °C group at 18 h post-feeding.	[87]
					12; 16; 20			Muscle concentrations declined faster at higher temperatures.	
			Florfenicol	10 mg·kg^−1^		10 days	Food	Florfenicol plasma and muscle concentrations were lower and depletion faster at higher temperatures.	
Antibiotic	*Carassius auratus gibelio*	Juvenile	Florfenicol	10 mg·kg^−1^	10; 25	5 days	Oral gavage	Elimination half-life of florfenicol from skin-on muscle and plasma was significantly higher at 10 °C.	[86]
								Metabolization of florfenicol was significantly higher at 25 °C.	
Antibiotic	*Carassius auratus gibelio*	Juvenile	Florfenicol	10 mg·kg^−1^	10; 20; 25	Single administration	Oral gavage	At higher water temperatures, florfenicol was absorbed, distributed, and eliminated more rapidly.	[62]
								Formation of florfenicol amine (metabolite) was faster at higher temperatures.	
Antibiotic	*Megalobrama ambycephala*	Juvenile	Florfenicol	25 mg·kg^−1^	18; 28	Single administration	Oral gavage	Maximum plasma concentrations of florfenicol were detected at 28 °C.	[81]
								Peak concentrations of florfenicol were reached significantly faster at 28 °C than at 18 °C.	
								Florfenicol elimination half-life in plasma, liver, kidney and muscle was significantly lower at 28 °C.	
Antibiotic	*Oreochromis niloticus*	Juvenile	Florfenicol	15 mg·kg^−1^	24; 28; 32	Single administration	Oral gavage	Elimination rate of florfenicol increased with temperature, more significantly at 32 °C.	[61]
							Intravenous injection	Elimination half-life and absorption half-life decreased in a temperature-dependent manner from 24 °C to 32 °C.	
								Maximum serum concentration of florfenicol decreased from 23.14 µg.mL^−1^ at 24 °C to 16.71 µg.mL^−1^ at 32 °C.	
								Time to reach maximum serum concentration decreased in a temperature-dependent manner.	
Antibiotic	*Carassius auratus gibelio*	Juvenile	Florfenicol	10 mg·kg^−1^	10; 20; 25	Single administration	Intramuscular injection	Elimination half-life of florfenicol decreased with the increase in temperature.	[85]
								Plasma concentration of florfenicol was always significantly higher at 10 °C.	
								Plasma concentration of florfenicol metabolite was highest at 25 °C, followed by 20 °C and 10 °C.	
Antibiotic	*Dicentrarchus labrax*	Juvenile	Danofloxacin	10 mg·kg^−1^	16; 27	5 days	Food	Elimination half-life of danofloxacin was significantly lower at 27 °C.	[84]
								Muscle danofloxacin concentration significantly higher at 10 °C (24 and 48 h post treatment).	
Antibiotic	*Pomatoschistus microps*	Juvenile	Cefalexin	1.3; 2.5; 5; 10 mg·L^−1^	20; 25	96 h	Water	Two highest cefalexin concentrations induced mortality at 20 °C; all concentrations induced mortality at 25 °C.	[88]
								Highest cefalexin concentration at 20 °C and the three highest concentrations at 25 °C reduced predatory performance	
								Cefalexin EC50 was lower at 25 °C compared to EC50 at 20 °C.	
Antibiotic	*Sparus aurata*	Juvenile	Oxytetracycline Flumequine Sulfadiazine Trimethoprim Oxanilic acid	30 mg·kg^−1^	14; 19.5	10 days	Food	All antibiotics except for trimethoprim had higher mean concentrations in muscle at 14 °C when compared to 19.5 °C.	[80]

								Elimination half-lives were lower at 19.5 °C for all antibiotics.	

								Elimination rates were lower at 14 °C for all antibiotics.	
Antibiotic	*Oreochromis niloticus*	Juvenile	Florfenicol	10 mg·kg^−1^	25; 30 20; 25 20; 25	10 days	Food	Elimination half-life of florfenicol was shorter in high temperatures than in low temperatures for the three fish species.	[82]
	*Sander vitreus*								
	*Hybrid striped bass (female white bass Morone chrysops × male striped bass M. saxatilis)*								
Antifungal	*Danio rerio*	Juvenile	Clotrimazole	2; 10 µg·L^−1^	28; 33	60 days	Water	Fish growth increased at 33 °C.	[95]
								Low clotrimazole concentration skewed development towards male differentiation at 33 °C.	
								High clotrimazole concentration skewed development towards male differentiation at both temperatures, but significantly more at 33 °C.	
Antifungal	*Micropogonias undulatus*	Juvenile/Adult	Triclosan	50 mg·kg^−1^	26; 29	10 days	Food	Exposure to triclosan at 29 °C caused higher bioaccumulation than at 26 °C.	[97]

## 4. Neuropsychiatric Drugs

Neuropsychiatric pharmaceuticals encompass a wide range of compounds such as antidepressants, anxiolytics, and mood stabilizers, among others. In Western societies, increasing prescription rates of anxiolytics and antidepressants have contributed to their occurrence, along with their metabolites, in water matrices at concentrations spanning ng·L^−1^ to µg·L^−1^ [21,48]. Consequently, there has been considerable debate regarding the impact of these types of drugs on fish, including their combined effects with temperature changes.

Anxiolytics modulate the interaction between gamma-amino acid butyric acid (GABA) and serotonin, while antidepressants inhibit the reuptake of serotonin, dopamine and noradrenaline from the presynaptic neuron, causing their persistence in the synaptic cleft and thus further stimulation of the postsynaptic neuron [99,100]. Since the components and mechanisms of synaptic transmission are highly conserved across evolution, including in fish [101,102], it is reasonable to suggest that these pharmaceuticals could disrupt neurological function in fish species. Zebrafish maintain strong homeostatic control of synaptic signaling across temperature ranges [102], while other fish models may show thermal sensitivity in neural excitability or metabolism. This variability may explain why certain behaviors (e.g., locomotor activity or predator avoidance) change with temperature and pharmaceutical exposure, while others (e.g., social or anxiety-related responses) remain relatively stable. Thus, the balance between excitatory and inhibitory signaling may shift with temperature, modifying how the nervous system responds to xenobiotic interference. Two notable examples of behavioral disruption by both an anxiolytic and an antidepressant have been reported with simultaneous temperature stress challenges (Table 3). The European perch (*Perca fluviatilis*) exposed to oxazepam at 10 °C and 18 °C exhibited an increased freezing behavior at the lower temperature and greater boldness with oxazepam at both temperatures, as evidenced by their movement from the test tank’s dark to light areas [103] (Table 3). Increased boldness and reduced predator avoidance may elevate predation risk in natural environments [104]. On the contrary, freezing responses or hypoactivity at lower temperatures may compromise foraging efficiency, decreasing energy acquisition and slowing growth. Therefore, oxazepam and temperature modulate fish behavior in ways that may reduce fitness in the wild, a risk likely to be exacerbated by rising global temperatures. In a study with guppies (*Poecilia reticulata*) exposed to fluoxetine under three different temperatures (18 °C, 24 °C and 32 °C), the antidepressant alone and the two highest temperatures stimulated mating behavior in males [105] (Table 3). However, no significant interaction was found between the two factors [105]. Increases in mating activity by fluoxetine and high temperatures may increase reproductive effort but this enhanced investment can also create energetic trade-offs by compromising immune function or parental care and ultimately lowering offspring survival [9]. In ecosystems already stressed by warming, the combined influence of higher temperature and neuropsychiatric pharmaceuticals could change population dynamics and reduce effective population size [4,18].

Fish pharmacokinetics and drug metabolism are also subject to changes caused by the interaction of antidepressants and anxiolytics and environmental temperature. The 28-day exposure of meagre (*Argyrosomus regius*) to the antidepressant venlafaxine at both 19 °C and 24 °C showed that at a higher temperature (24 °C), the water uptake of this pharmaceutical was reduced and the biotransformation and export from the liver and muscle increased (Table 3) [106]. However, at 24 °C the brain uptake of venlafaxine was higher than at 19 °C [106]. Venlafaxine is designed to act specifically on the central nervous system [107], thus the increased brain uptake could in fact exacerbate the negative effects in fish, under a global warming scenario. A similar pattern was observed in European perch (*Perca fluviatilis*) exposed to the anxiolytic temazepam under two temperature regimes of 10 °C and 20 °C (Table 3) [108]. Although the parental compound temazepam was more abundant in tissues at 10 °C, its metabolization into oxazepam was greater at 20 °C. As a result, this biologically active metabolite was found in higher concentrations in the brain and liver at higher temperature (Table 3), which could ultimately lead to stronger or novel effects that oxazepam may induce in perch. A different study analyzed the combined effects of venlafaxine and temperature, by exposing zebrafish to this antidepressant at 27 °C and 32 °C [109]. It was found that both miRNA-22b and miRNA-301a, which target a number of different pathways, namely RNA degradation and amino acid biosynthesis, were significantly downregulated by the venlafaxine and high temperature, both individually and combined (Table 3) [109].

Carbamazepine is an anticonvulsant pharmaceutical used to treat seizures, neuropathic pain and, in some cases, bipolar disorder [110]. Two previously described studies [73,74] used Senegalese sole as a model organism to evaluate the effects of carbamazepine (Table 3) exposure via intraperitoneal injection at 15 °C and 20 °C. The results from Aceña et al. [74] demonstrated a negative effect of temperature, with hepatic enzymatic activity only being traceable at the lower temperature regime of 15 °C. González-Mira et al. [73] found a tendency for the effects of the carbamazepine injection to be more severe at 20 °C when compared to 15 °C, namely in the expression of heat-shock protein 70 (HSP70), the activity of certain enzymes or a reduction in plasma triglyceride concentration (Table 3).

**Table 3:** Summary of methodology and effects of combined exposure to temperature and psychiatric or neurological pharmaceuticals.

Therapeutic Class	Species	Life Stage	Pharmaceutical(s)	Dose	Temperature (°C)	Exposure Period	Administration	Effects	Reference
Antidepressant	*Oreochromis niloticus*	Adult (In vitro)	Venlafaxine	0.001; 0.01; 0.1; 1; 10 µg·L^−1^	25; 35; 40; 45	1 h	Water	Mitochondrial respiratory rate was lower at 35, 40 and 45 °C.	
								Respiratory complexes I, II and IV were severely depressed by venlafaxine at 45 °C.	[67]
								Succinate dehydrogenase activity increased with temperature.	
								Cytochrome C oxidase activity decreased at 45 °C.	
Anxiolytic	*Perca fluviatilis*	Juvenile	Temazepam Oxazepam (metabolite)	0.2; 2.0 µg·L^−1^	10; 20	8 days	Water	Higher bioconcentration of temazepam in the brain at 10 °C.	[108]
								Higher bioconcentration of oxazepam in the brain and liver at 20 °C.	
								Temazepam metabolization (into oxazepam) higher at 20 °C.	
Antidepressant	*Danio rerio*	Adult	Venlafaxine	1 µg·L^−1^	27; 32	21 days	Water	Decrease relative abundance of miR-22b-3p and miR-301a by venlafaxine, high temperature and venlafaxine plus temperature.	[109]
Antidepressant	*Poecilia reticulata*	Adult	Fluoxetine	38; 312 ng·L^−1^	18; 24; 32	15 months	Water	Males kept at 18 °C had fewer copulation attempts and courting behaviors.	[105]
								Less active fish at 18 °C.	
								Increased male copulation by fluoxetine.	
								Decreased reproductive behaviors in males at 18 °C.	
								No interaction effects between fluoxetine and temperature.	
Anxiolytic	*Perca fluviatilis*	Juvenile	Oxazepam	10 µg·L^−1^	10; 18	7 days	Water	Oxazepam increased bold behavior.	[103]
								Freezing behavior increased at 10 °C. No interaction between oxazepam and temperature.	
Antidepressant	*Argyrosomus regius*	Juvenile	Venlafaxine	20 µg·L^−1^; 160 µg·kg^−1^	19; 24	28 days	Water and Food	Reduced venlafaxine uptake via water at 24 °C.	[106]
								Higher venlafaxine elimination from the liver and muscle at 24 °C.	
								Brain uptake increased at 24 °C.	
Anticonvulsant	*Solea senegalensis*	Juvenile	Carbamazepine	1 mg·kg^−1^	15; 20	48 h	IP Injection	CYP and carboxylesterase activities were traceable only at 15 °C.	[74]
Anticonvulsant	*Solea senegalensis*	Juvenile	Carbamazepine	1 mg·kg^−1^	15; 20	48 h	IP Injection	Carbamazepine at 20 °C significantly reduced plasma triglycerides.	[73]
								BFCOD activity increased after drug administration at 15 °C.	
								HSP70 was significantly elevated after carbamazepine administration at 20 °C.	
								UDPGT activity decreased more severely at 20 °C than at 15 °C after drug administration.	

## 5. Estrogenic Hormones

Due to the extensive use of estrogenic hormones in oral contraceptives and hormone replacement therapies [111,112,113], these compounds are widely detected in aquatic environments, usually at the ng·L^−1^ range [33,34,114,115]. Their potential to disrupt endocrine function in fish has been the focus of significant research over the years, with studies reporting effects of estrogenic exposure that include low sperm mobility and gonadosomatic index (GSI) in males [116], gonadal feminization in males [117,118,119], changes in behavior [120,121] and vitellogenin induction [120,122,123]. This has resulted in a comprehensive body of literature that describes typical biological responses, such as the well-characterized induction of vitellogenin synthesis, a biomarker of estrogenic activity [124,125].

In the context of combined effects with warming water temperature scenarios, estrogenic pharmaceuticals emerged as the second most frequently investigated class (Table 4). Most studies focused on assessing alterations in recognized indicators of estrogenic activity across a range of temperature conditions. A significant increase in estrogenic response at higher temperatures (19 °C), namely a stronger increase in vitellogenin mRNA expression, was reported after the simultaneous exposure of 17α-ethinylestradiol (EE2) at 12 °C and 19 °C in juvenile brown trout (*Salmo trutta*) for 21 days (Table 4) [126]. A study using adult fathead minnows (*Pimephales promelas*) found a similar pattern profile: plasmatic vitellogenin was significantly increased in individuals exposed to estrone (E1) at the higher temperature regime of 26 °C compared to the lower temperature of 18 °C (Table 4) [127]. However, there are contradictory results from two other studies with estrogenic inputs plus high-temperature conditions. Adult fathead minnows exposed to E1 under four temperature regimes that simulate seasonal variations (15, 18, 21 and 24 °C) showed increased plasma vitellogenin concentration in males when exposed to E1 alone. However, at 24 °C this effect was reversed, yielding plasma vitellogenin levels that were markedly lower than those observed at the other temperatures [128] (Table 4). Similarly, bluegill sunfish (*Lepomis macrochirus*) exposed to E1 under the same seasonal temperature regime (15, 18, 21 and 24 °C) had lower plasma vitellogenin levels at 24 °C when compared to all other temperatures and the controls [129] (Table 4). In both cases, there was evidence of estrogenic effects—such as increased liver weight, gonadosomatic index (GSI) and hepatosomatic index (HSI)—being minimized or abolished by higher temperatures (Table 4). The reduction in plasma vitellogenin levels at 24 °C may result from several factors, likely including an increase in the metabolization of E1, which could lower the endogenous hormone levels and consequently its effects. Alternatively, or additionally, the increase in metabolic rates at higher temperatures [18] may redirect energy away from processes such as vitellogenin synthesis towards coping with thermal stress.

The temperature regimes and estrogenic inputs also interfere with the process of fish growth. In juvenile zebrafish reared at 23 °C, 28 °C, or 33 °C, growth was accelerated under higher temperature regimes, and the synthetic estrogen EE2 exacerbated this effect [130]. The same trend was observed in fathead minnow larvae exposed to E1 at four different temperatures (15, 18, 21 and 24 °C) to represent seasonal variations, with the ones reared at 24 °C exhibiting higher body length [131] (Table 4), which could be a consequence of an increase in basal metabolic rates [11]. However, Ward et al. [131] noted that E1 impaired fish growth, particularly at 15 °C (Table 4). Another study with female Japanese flounder (*Paralichthys olivaceus*) exposed to 17β-estradiol (E2) at either 18 or 27.5 °C also found that the larvae reached higher body lengths at higher temperatures and that E2 had an adverse effect on growth [132]. Additionally, estrogens and temperature changes could also greatly disrupt fish sexual differentiation. The environmental temperature can influence the sexual development of fish and the sex ratios of populations [94], as shown by rearing pejerrey (*Odontesthes bonariensis*) at 17, 25, or 29 °C, which resulted in 0%, 55.2% and 100% male populations, respectively [133]. This study also found that the expression of *anti-Müllerian hormone* (*amh*), a gene linked to male sexual development [134], peaked at 29 °C but was downregulated after exposure to E2 [133] (Table 4). In the female Japanese flounder larvae, the expression of *amh* was significantly higher at 27.5 °C, and the originally female larvae developed sperm ducts and compact spermatogonia in their gonads at 75 and 120 days post-hatching, respectively [132] (Table 4). The opposite effect was observed in zebrafish, which had accelerated sexual development when exposed to EE2 at lower temperatures; however, estrogen stunted male differentiation at a 33 °C regime [130] (Table 4). In juvenile European sea bass (*Dicentrarchus labrax*) exposed to female- or male-inducing temperatures (15 and 21 °C, respectively) and/or E2 during their hormonally sensitive period for sexual differentiation, the high temperature alone generated a population with 78% males, whilst the E2 was able to induce total feminization at 21 °C [135] (Table 4). Research indicates that for fish exhibiting temperature-dependent sexual differentiation, the ongoing increase in water temperatures may significantly bias populations towards males, even with only a 1–2 °C rise [136], which is below current model projections [1]. The ability of estrogens, such as E2, to induce complete feminization in fish [135,137] demonstrates how the interplay of pharmaceuticals—especially estrogenic hormones—and rapidly increasing water temperatures may threaten population sustainability. Over successive generations, these processes can diminish genetic diversity and cause population imbalances [4,18].

**Table 4:** Summary of methodology and effects of combined exposure to temperature and estrogenic pharmaceuticals.

Therapeutic Class	Species	Life Stage	Pharmaceutical	Dose	Temperature (°C)	Exposure Period	Administration	Effects	Reference
Natural Estrogen	*Lepomis macrochirus*	Adult	Estrone (E1)	90; 414 ng·L^−1^	15; 18; 21; 24	30 days	Water	Male condition factor was higher at 18 °C.	[129]
								Male livers of fish kept at 15 °C were larger.	
								Males exposed to E1 had higher plasma vitellogenin levels compared to the control.	
								Plasma vitellogenin was lower in males exposed to E1 at 24 °C.	
								Females kept at 15 °C had significantly larger livers than those kept at 21 °C.	
Natural Estrogen	*Pimephales promelas*	Adult	Estrone (E1)	12.5; 25; 65 ng·L^−1^	15; 18; 21; 24	30 days	Water	Male plasma vitellogenin was elevated by E1 treatment and decreased with the rise in temperature.	[128]
								Exposure of males to 65 ng.L^−1^ of E1 caused significant decrease in the hematocrit.	
								Lower temperatures caused higher gonad weight, GSI, liver weight and blood glucose in males.	
								Females exposed to 65 ng.L^−1^ of E1 showed a decrease in hematocrit.	
								Females kept at lowers temperatures had higher liver vacuolization, gonad weight, GSI, HSI and sexual maturity.	
Natural Estrogen	*Pimephales promelas*	Adult	Estrone (E1)	9; 14; 78; 135 ng·L^−1^	18; 26	28 days	Water	Fish kept at 26 °C had higher mortality rate and decreased secondary sexual characteristics.	[127]
								High temperature caused higher GSI and lower testis weight.	
								E1 exposure increased testis weight.	
								Higher concentrations of E1 and higher temperature increased plasma vitellogenin.	
Natural Estrogen	*Pimephales promelas*	Adult/Larvae	Estrone (E1)	5; 25; 125 ng·L^−1^	15; 18; 21; 24	30 days	Water	Larval growth was higher at higher temperatures.	[131]
								Escape behavior had higher latency with higher temperatures and velocity of escape was negatively correlated with temperature rise.	
								Prey capture success increased in a temperature-dependent manner and was negatively affected by E1 exposure at 15 °C.	
								Prey capture rate increased in a temperature-dependent manner.	
								Male adults kept at 21 °C had more aggressive behavior.	
								Hatching latency increased with temperature, but at 18, 21 and 24 °C it was lowered by E1 relative to the respective temperature control.	
								E1 lowered body length at all temperatures at 15 °C.	
								At 15 °C, E1 caused a decrease in larval survival.	
Natural Estrogen	*Paralichthys olivaceus*	Larvae	17β-estradiol (E2)	8 mg·kg^−1^	18; 27.5	90 days (from 30 to 120 days post-hatching)	Food	Larvae reared at 27.5 °C had the highest body length.	[132]
								Larvae reared with E2 had lower body length compared to control larvae.	
								Increased *anti-müllerian hormone* (*amh*) expression at 27.5 °C and E2.	
								Increased *cytochrome 19a1a* expression rose over time in control and E2 groups.	
								At 75 dph, larvae gonads had sperm ducts at 27.5 °C.	
								At 120 dph, heat treatment (27.5 °C) had numerous and compact spermatogonia.	
Natural Estrogen	Dicentrarchus labrax	Juvenile	17β-estradiol (E2)	10 mg·kg^−1^	15; 21	200 days	Food	Fish reared at 21 °C with E2 were 100% female, whereas only 21% of the fish reared at 21 °C alone were female.	[135]
								Fish exposed to E2 did not differ in sex ratio at both 15 and 21 °C.	
								After 170 days, fish exposed to E2 at 21 °C were smaller than control fish at 21 °C.	
								Females reared at 21 °C had higher GSI when compared to females reared at the same temperature with E2.	
								E2 exposure downregulated *amh*, *tescalcin* (*tesc*) and *steroidogenic acute regulatory protein* (*star*) mRNA.	
Natural Estrogen	*Odontesthes bonariensis*	Larvae	17β-estradiol (E2)	50 µg·g^−1^	17; 25; 29	8 weeks	Food	There were 0% males at 17 °C, 55.2% males at 25 °C and 100% males at 29 °C.	[133]
								*Amh* expression peaked significantly higher at 29 °C.	
								*Amh* expression in females feminized with E2 was downregulated.	
Synthetic Estrogen	*Gasterosteus aculeatus*	Embryo/Larvae	17α-ethinylestradiol (EE2)	15 ng·L^−1^	13 to 23	32 days	Water	Egg diameter, embryo and larval survival rates and standard length were reduced by higher temperatures in the presence of EE2.	[138]
								EE2 caused partial feminization of testis and upregulation of estrogen receptors.	
								EE2 at 29 °C caused the lowest body weight.	
Synthetic Estrogen	*Poecelia reticulata*	Adult	17α-ethinylestradiol (EE2)	5 ng·L^−1^	26; 29	45 days	Water	Hepatic lipid droplets were depleted by EE2 at 29 °C.	[13]
								EE2 reduced HSI, liver volume, hepatocyte volume and nuclear volume.	
Synthetic Estrogen	*Danio rerio*	Juvenile	17α-ethinylestradiol (EE2)	4 ng·L^−1^	23; 28; 33	60 days	Water	Fish raised at 23 °C were smaller, and fish raised at 33 °C were larger.	[130]
								Fish raised under EE2 exposure were larger than the corresponding temperature control at 23 °C and 33 °C.	
								EE2 exposure at 23 °C accelerated sexual differentiation.	
								Fish kept at 33 °C were more developed in both males and females, but EE2 exposure delayed male gonad development.	
Synthetic Estrogen	*Salmo trutta*	Juvenile	17α-ethinylestradiol (EE2)	3 ng·L^−1^	12; 19	21 days	Water	HSI was lower in the fish exposed to EE2 at 12 °C.	[126]
								EE2 exposure upregulated *vitellogenin A* (*VtgA*) mRNA.	
								*VtgA* upregulation was higher at 19 °C and *estrogen receptor α* (*ERα*) upregulation by EE2 was only found at 19 °C.	

## 6. Discussion and Conclusions

**Figure 2:**
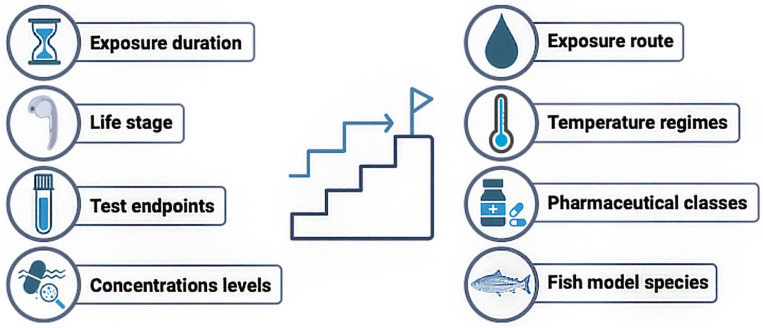
Future recommendations for assessing the combined effects of pharmaceutical pollution and global warming in fish.

This review synthesizes and critically analyzes the current available literature on the combined effects of pharmaceutical exposure and temperature stress on fish, considering the increasing influx of pharmaceuticals into aquatic environments under scenarios of global warming and associated rising water temperatures. A systematic search across PubMed, Web of Science, and Scopus identified only 39 peer-reviewed studies meeting the inclusion criteria (Supplementary Table S1), underscoring a substantial knowledge gap. This is particularly concerning, given the projections of severe climate change impacts [1] and the parallel and well-documented potential of pharmaceuticals to disrupt aquatic organisms [51]. Several aspects of the reviewed literature warrant deeper examination and structured synthesis. Accordingly, for each of the following topics, we provide a summary of the key findings and knowledge gaps. A clear and prescriptive summary with emerging research directions is provided in Figure 2.

Before drawing any firm conclusions, it is essential to acknowledge several limitations in the current published research, despite the reviewed studies providing insightful information about the combined effects of pharmaceuticals and warmer temperatures on fish. Many studies were conducted with relatively small sample sizes (likely due to the ethical constraints and costs involved in increasing the number), or short exposure times, which might not accurately reflect long-term or ecological realities. Due to differences in species, life stages, dosages, and experimental setups, direct comparison of results is challenging. All studies were conducted under highly controlled laboratory conditions, which are not representative of real-world circumstances. Overall, to better understand and predict the risks that fish populations will face in a warming and increasingly contaminated world, these factors highlight the need for more standardized and ecologically relevant approaches, namely considering multiple stressors and chronic exposures. Despite the described restrictions, conclusions can still be drawn.

### 6.1. Pharmaceutical Classes

Antibiotics were the most represented class of pharmaceuticals in this review, representing 41% of all studies. They are among the three most prescribed pharmaceutical classes worldwide [22] and are of particular concern due to their persistence, incomplete metabolization [139], and their contribution to antibiotic resistance in microorganisms, an increasing trend [140]. In 2021, antibiotic production in China was estimated at 92,700 tons, with over half destined for livestock [141]. A significant proportion of the parental forms or metabolites of those antibiotics enter aquatic environments, particularly due to their widespread use in aquaculture [142,143]. The use in aquaculture production has motivated over 75% of the antibiotic studies identified in this review, most of which examined how the pharmacokinetics of antibiotics change across temperatures under a range that represents seasonal fluctuations. Notably, florfenicol, a widely used antibiotic in aquaculture [144,145,146], was used in over 20% of the antibiotic studies reviewed. A smaller proportion of studies—approximately 19%—investigated the toxic effects in fish caused by antibiotics plus temperature, rather than their pharmacokinetics. The toxicity of antibiotics within a global warming scenario is a topic that needs further investigation given the potential of antibiotics to harm fish [77,78,79].

Estrogenic hormones were the second most represented pharmaceutical class (28.2% of the reviewed studies). Although they are not among the top three most prescribed classes [22], endocrine-disrupting compounds—including estrogenic hormones—are widely used. The most notable pharmaceutical application is in oral contraceptives [147], but estrogens are also prescribed for other purposes, such as osteoporosis treatment [148]. The effects of estrogens on fish have long been studied, particularly in relation to their impact on reproduction [116,117,119,120,122]. In our dataset, 73% of studies assessing estrogens focused on reproductive endpoints (e.g., plasma vitellogenin, gonadal development), while 27% examined the impacts of estrogenic hormones on sexual differentiation at different temperatures. This is relevant because sexual differentiation in many fish species is plastic and influenced by environmental conditions [149].

Neuropsychiatric pharmaceuticals ranked third, accounting for approximately 21% of the studies. These medicines are commonly used, and their consumption has been steadily increasing over the last decade [150,151]. Over 70% of these studies analyzed the effects of anxiolytics or antidepressants, focusing on bioaccumulation patterns or behavioral changes under different temperature regimes. Given the evolutionary conservation of neurotransmission pathways, these pharmaceuticals have a high likelihood of causing significant effects in fish, particularly antidepressants that inhibit serotonin reuptake, given serotonin’s broad physiological roles [152]. Carbamazepine, an anticonvulsant [110], undergoes hepatic biotransformation via cytochrome P450 enzymes in mammals [52]. This makes the two studies that investigated its effects in fish particularly relevant, as they examined how hepatic biotransformation activity varied under different temperature regimes [73,74].

Analgesics and anti-inflammatories were underrepresented, accounting for only 10.3% of the studies—considerably fewer than other classes. Despite this, these drugs are among the three most widely prescribed worldwide [22], representing the largest-selling class of pharmaceuticals in 2017 [153]. Their limited representation in temperature–pharmaceutical interaction research highlights the need for further studies examining their combined effects in the context of projected global warming scenarios.

Finally, several pharmaceutical classes were absent from the literature on combined effects with temperature. Among these hypolipidemic pharmaceuticals, such as fibrates and statins, have gained worldwide popularity in recent years, with consumption nearly quadrupling between 2000 and 2017 [153], primarily for treating dyslipidemias [154]. Their impacts on fish have already been documented, including changes in lipid homeostasis [155,156] and developmental effects [157]. However, to our knowledge, no studies have checked their combined impacts with elevated temperatures, representing a clear gap in understanding the potential risks these pharmaceuticals may cause under climate change scenarios.

### 6.2. Temperature Range and Exposure Duration

When reviewing the experimental designs, the range of temperature regimes emerged as a critical aspect. Regarding the tolerance limits of each fish species, each study tested distinct temperature regimes, depending on its primary aim. Of the 39 papers, 16 of them (41%) simulated seasonal variations to which species were naturally exposed. These studies combined seasonal temperatures with either antibiotics or estrogenic hormones to analyze the pharmacokinetics of specific antibiotics across seasons in aquaculture or the effects of estrogenic inputs at different times of the year. Environmental temperature has a strong influence on fish metabolism [2,3], reproduction [94], and pharmaceutical metabolism, making such approaches particularly relevant, for example, in determining the appropriate administration of antibiotics in aquaculture [158].

Only ten papers (25.6%) exposed fish to their ideal temperature and to a level 5 °C higher, simulating worst-case global warming predictions [1]. These studies encompassed various pharmaceutical classes, including anti-inflammatories, antifungals, antibiotics, and neuropsychiatric medications. Despite this, there is still a lack of research focusing on temperature scenarios that mimic global warming. As environmental temperatures rise, fish are expected to move towards preferred ranges [5,6], though physical barriers may constrain this response, especially in river systems [9].

Four of the papers (10.3%) focused on sexual differentiation, combining estrogenic hormones with temperatures known to influence sex ratios, with higher and lower temperatures promoting masculinization or feminization. Those temperatures were species-specific and based on existing literature rather than global warming projections. Lastly, eight of the studies (20.5%) tested temperatures within species tolerance ranges that did not reflect seasonal or projected warming, aiming instead to assess toxicity or characterize pharmacokinetics.

Exposure duration also varied across investigations, ranging from acute to chronic, depending on endpoints. No clear link to a pharmaceutical class was found, although noticeable trends emerged. Acute exposures (<15 days) predominated, occurring in 56.4% of the publications. All antibiotic studies were acute assays, including single administrations, and consistently focused on pharmacokinetics (see Section 6.1). Sub-chronic (15–90 days) and chronic (>90 days) exposures accounted for 35.9% and 7.7%, respectively. Estrogenic hormone studies were exclusively sub-chronic or chronic, reflecting their focus on sexual differentiation. In the context of global warming and pharmaceutical pollution, although heat waves may last only a few days, longer exposures may better reflect environmental conditions and are likely more informative regarding the dual disruptive effects of pharmaceuticals and rising temperatures.

### 6.3. Species and Development Stage

The interaction between warming and pharmaceutical exposure is highly species-dependent, underscoring the need for testing diverse fish species. The 39 studies reviewed utilized 25 different species, comprising 15 freshwater and 10 marine fish. Representation of both groups is critical, as warming and pollution pressures differ across aquatic environments, with freshwater systems being generally more vulnerable [9]. Zebrafish (12.8%) was the most common model, followed by Nile tilapia (10.2%), fathead minnow, and Prussian carp (7.7% each). Fish models included tropical, subtropical, and temperate latitudes, reflecting broad geographical distributions. This diversity is also critical given that warming affects differently across latitudes, with higher latitudes warming faster [159] and potentially affecting local fish populations more severely.

The fish life stage also affects the model’s sensitivity to pharmaceutical and warming effects. Larvae and juveniles are generally more vulnerable than adults. Over 60% of the studies used juveniles, while only 15% tested larvae. This bias reflected the focus of antibiotic and estrogenic studies: antibiotics mainly were tested in aquaculture species, where juveniles dominate production, and estrogenic studies targeted sexual differentiation, which requires immature life stages. It is important that future studies encompass all life stages, to provide the broadest and clearest picture of the impacts of pharmaceuticals and warming scenarios. Lastly, in vitro studies were not included in the present review, but they could provide greater insights into the underlying mechanisms of temperature-pharmaceutical interaction. The use of in vitro and in vivo methodologies in tandem would provide the most comprehensive approach to the study of the impacts of pharmaceutical pollution in ever-warming aquatic environments.

### 6.4. Future Perspectives

Future research investigating the combined impacts of pharmaceutical pollution and global warming on wild fish populations urgently requires standardized guideline assays, harmonized methodological approaches, and stronger integration of ecological relevance. Without these measures, research data will lack comparability, outputs will remain limited, and scientific progress in this critical area will be hampered by the absence of reliable and high-quality datasets needed to support global policy decisions. In our view, to advance this field, the following key considerations should be implemented (Figure 2):

(i) Standardization of exposure durations. Future studies should prioritize sub-chronic and chronic exposures, which better reflect environmentally relevant conditions and allow for a better understanding of population-level impacts.

(ii) Systematization of testing across life stages. It is important to develop a framework to match the life stage to specific endpoints. For instance, embryos and larvae are better suited for the study of developmental or teratogenic endpoints, whereas juveniles are prime candidates for growth studies and mature adults would be used for reproductive research. Early life stages are usually more vulnerable to environmental stressors, so this should also be taken into consideration by test protocols.

(iii) Standardization of endpoints. According to the specific study objective (i.e., reproduction, development, growth), specific and standardized endpoints should be defined. Additional endpoints could be added to the standardized ones.

(iv) Exposure to environmentally relevant concentrations. Exposure levels must mimic a mean environmental concentration, typically in the ng·L^−1^–µg·L^−1^ range, as these better represent real environment scenarios. Single and mixture exposures should be considered. It is also important to monitor and maintain the concentration levels throughout the exposure periods.

(v) Prioritizing waterborne exposure routes. Wild fish populations are primarily exposed to dissolved or particulate pharmaceuticals through the water; as such, waterborne exposures should be the standard. Dietary, gavage or injection administrations should be reserved for exploratory, mechanistic, or pharmacokinetic studies. In particular, dietary foods could be specifically suitable for certain species (e.g., flatfish) or in aquaculture settings. 

(vi) Standardization of temperature regimes. Studies should include a standard temperature for the fish species and at least a “worst-case” warming scenario (+5 °C) according to prediction models [1]. Ideally, an “intermediate” warming scenario should also be tested.

(vii) Selection of pharmaceuticals or therapeutic classes for tests. The criteria for selection should include current data from up-to-date environmental guidelines [160] and monitoring reports, targeting classes frequently detected [161]. The strategy should also include under-studied yet widely prescribed drugs (e.g., hypolipidemic or antidiabetic drugs) to fill critical knowledge gaps.

(viii) Establish a core of priority species to use as models. Since freshwater and marine environments differ in susceptibility to warming and pharmaceutical pollution (with freshwater and riverine systems usually at higher risk), species should be selected to represent both environments. Moreover, since global warming affects temperatures differently across latitudes, the model species should include representatives from higher and lower latitudes in both freshwater and marine systems.

Collectively, these directives should enable the generation of more reproducible and comparable data, whilst drawing the experimental settings closer to actual environmental conditions.

## Data Availability

No new data were created in this study. Data sharing is not applicable to this article.

## Supplementary Materials

The following supporting information can be downloaded at: https://www.mdpi.com/article/10.3390/jox15060190/s1, Table S1: Accepted peer-reviewed papers that met the inclusion criteria.
