# Supplementary material for: A Double Challenge for Fish: The Combined Stress of Warming and Pharmaceuticals in Aquatic Systems
Source: J Xenobiot. 2025 Nov 8;15(6):190. doi: 10.3390/jox15060190 (PMC12641760; doi:10.3390/jox15060190)
Supplement: Supplementary file 1 [file jox-15-00190-s001.zip › jox-3908140-supplementary.pdf]

Supplementary Materials: A Double Challenge for Fish: The Combined Stress of Warming and Pharmaceuticals in Aquatic Systems

Tiago Lourenço, Maria João Rocha, Eduardo Rocha and Tânia Vieira Madureira

Supplementary Table S1. Accepted peer-reviewed papers that met the inclusion criteria.

Therapeutic Class	Species	Life Stage	Pharmaceutical(s)	Temperature (ºC)	Exposure Period	Reference
Analgesic<br><br>Anti-inflammatory	*Danio rerio*	Larvae	Paracetamol<br><br>Diclofenac	28; 33	78 hours	Boreham et al., (2024)
Analgesic	*Oryzias latipes*	Larvae	Paracetamol	15; 25; 30	4 days	Kataoka et al., (2019)
Anti-inflammatory<br><br>Anticonvulsant	*Solea senegalensis*	Juvenile	Ibuprofen<br><br>Carbamazepine	15; 20	48 hours	Aceña et al., (2017)
Anti-inflammatory<br><br>Anticonvulsant	*Solea senegalensis*	Juvenile	Ibuprofen<br><br>Carbamazepine	15; 20	48 hours	González-Mira et al., (2016)
Antibiotic	*Micropterus salmoides*	Juvenile/Adult	Enrofloxacin<br><br>Ciprofloxacin	17; 22; 27	5 days	Fan et al., (2026)
Antibiotic	*Oryzias malastigma*	Embryos	Florfenicol	20; 25; 30	21 days	Guo et al., (2025)
Antibiotic	*Danio rerio*	Juvenile	Trimethoprim<br><br>Sulfamethoxazole	26; 28; 32	28 days	Diogo et al., (2025)
Antibiotic	*Sebastes schlegelii*	Adult	Oxolinic acid	13; 22	Single dose	Bae et al., (2025b)
Antibiotic	*Sebastes schlegelii*	Juvenile	Enrofloxacin<br><br>Ciprofloxacin	13; 22	Single dose	Bae et al., (2025a)
Antibiotic	*Oreochromis niloticus*	Juvenile	Amoxicillin	25; 30	5 days	Rairat et al., (2024)

Antibiotic	*Salmo salar*	Juvenile	Tetracycline<br>Florfenicol	12; 16; 20	15 days<br>10 days	Zanuzzo et al., (2022)
Antibiotic	*Carassius auratus gibelio*	Juvenile	Florfenicol	10; 25	5 days	Yang et al., (2020a)
Antibiotic	*Carassius auratus gibelio*	Juvenile	Florfenicol	10; 20; 25	Single administration	Yang et al., (2019)
Antibiotic	*Megalobrama ambycephala*	Juvenile	Florfenicol	18; 28	Single administration	Huang et al., (2019)
Antibiotic	*Oreochromis niloticus*	Juvenile	Florfenicol	24; 28; 32	Single administration	Rairat et al., (2019)
Antibiotic	*Carassius auratus gibelio*	Juvenile	Florfenicol	10; 20; 25	Single administration	Yang et al., (2018b)
Antibiotic	*Dicentrarchus labrax*	Juvenile	Danofloxacin	16; 27	5 days	Vardali et al., (2017)
Antibiotic	*Pomatoschistus microps*	Juvenile	Cefalexin	20; 25	96 hours	Fonte et al., (2016)
Antibiotic	*Sparus aurata*	Juvenile	Oxytetracycline<br>Flumequine<br>Sulfadiazine<br>Trimethoprim<br>Oxanilic acid	14; 19.5	10 days	González et al., (2010)
Antibiotic	*Oreochromis niloticus*<br>*Sander vitreus*<br>*Hybrid striped bass (female white bass Morone chrysops × male striped bass M. saxatilis)*	Juvenile	Florfenicol	25;30<br>20;25<br>20;25	10 days	Kosoff et al., (2009)
Antifungal	*Danio rerio*	Juvenile	Clotrimazole	28; 33	60 days	Brown et al., (2015)

Antifungal	*Micropogonias undulatus*	Juvenile/Adult	Triclosan	26; 29	10 days	Hedrick-Hopper et al., (2015)
Antidepressant	*Oreochromis niloticus*	Adult (In vitro)	Venlafaxine	25; 35; 40; 45	1 h	Borowiec et al., (2025)
Anxiolytic	*Perca fluviatilis*	Juvenile	Temazepam<br><br>Oxazepam (metabolite)	10; 20	8 days	Cerveny et al., (2021)
Antidepressant	*Danio rerio*	Adult	Venlafaxine	27; 32	21 days	Ikert and Craig (2020)
Antidepressant	*Poecilia reticulata*	Adult	Fluoxetine	18; 24; 32	15 months	Wiles et al., (2020)
Anxiolytic	*Perca fluviatilis*	Juvenile	Oxazepam	10; 18	7 days	Saaristo et al., (2019)
Antidepressant	*Argyrosomus regius*	Juvenile	Venlafaxine	19; 24	28 days	Maulvault et al., (2018)
Natural Estrogen	*Lepomis macrochirus*	Adult	Estrone (E1)	15; 18; 21; 24	30 days	Korn et al., (2020)
Natural Estrogen	*Pimephales promelas*	Adult	Estrone (E1)	15; 18; 21; 24	30 days	Cox et al., (2018)
Natural Estrogen	*Pimephales promelas*	Adult	Estrone (E1)	18; 26	28 days	Shappell et al., (2018)
Natural Estrogen	*Pimephales promelas*	Adults and larvae	Estrone (E1)	15; 18; 21; 24	30 days	Ward et al., (2017)
Natural Estrogen	*Paralichthys olivaceus*	Larvae	17β-Estradiol (E2)	18; 27.5	90 days (from 30 to 120 days post-hatching)	Yang et al., (2020b)
Natural Estrogen	Dicentrarchus labrax	Juvenile	17β-Estradiol (E2)	15; 21	200 days	Días and Piferrer (2017)
Natural Estrogen	*Odontesthes bonariensis*	Larvae	17β-Estradiol (E2)	17; 25; 29	8 weeks	Fernandino et al., (2008)
Synthetic Estrogen	*Gasterosteus aculeatus*	Embryo/Larvae	17α-ethinylestradiol (EE2)	13 to 23	32 days	Devergne et al., (2025)

Synthetic Estrogen	*Poecelia reticulata*	Adult	17α-ethinylestradiol (EE2)	26; 29	45 days	Vilaça et al., (2025)
Synthetic Estrogen	*Danio rerio*	Juvenile	17α-ethinylestradiol (EE2)	23; 28; 33	60 days	Luzio et al., (2016)
Synthetic Estrogen	*Salmo trutta*	Juvenile	17α-ethinylestradiol (EE2)	12; 19	21 days	Korner et al., (2008)
